# Characterization of Microsatellite Loci in the Western Subterranean Termite, *Reticulitermes hesperus*, and Cross-Amplification in Closely Related Cryptic Species

**DOI:** 10.1673/031.007.1701

**Published:** 2007-03-29

**Authors:** Kirsten A. Copren

**Affiliations:** Center for Population Biology, Department of Entomology, University of California, Davis, CA 95616 Current address: Genome Analysis Core, Comprehensive Cancer Center, University of California, 2340 Sutter Street, San Francisco, CA 94115

**Keywords:** Isoptera, cryptic species, population structure

## Abstract

New, and previously reported microsatellites, were characterized for a group of four cryptic sibling species in California (USA) in the subterranean termite genus *Reticulitermes* with the goal of finding loci appropriate for population and species level studies. Three new microsatellites were identified originating from *R. hesperus*, and 19 loci previously characterized in *R. flavipes* and *R. santonensis* were examined. Of the three loci specifically developed for *R. hesperus*, none amplify with the other species. Variation appropriate for population level studies was found in 4–13 loci depending on the species. Fifteen loci appeared to be appropriate for use at the species level. Unique or monomorphic alleles are found among the four species, indicating these loci will be taxonomically informative for this group.

## Introduction

*Reticulitermes* is a genus of economically important termite species found in temperate climates of the Holarctic (Weesner 1970). A recent phylogenetic analysis using mtDNA sequence data (Copren et al. 2005) supported the identification of cryptic sibling species proposed based on chemotaxonomic data (Haverty and Nelson 1997). Correct characterization of the population structure and taxonomy of these species is required for proper control and to better understand their evolutionary history. Due to their cryptic behavior, basic biological information regarding colony and population structure has been difficult to obtain in the genus *Reticulitermes*. Genetic studies have been helpful in understanding population and species-level structure. Notably, genetic studies using mitochondrial DNA on the eastern North American *R. flavipes* and European *R. santonensis* support the synonomy of these two species (Jenkins et al. 2001; Austin et al. 2005). Studies using microsatellites developed for these species have been particularly helpful in understanding population and colony genetic structure and breeding systems as well (Vargo 2003; Dronnet et al. 2005). However, no similar studies using microsatellites have been performed on western North American species, which have been further confounded due to the presence of cryptic species.

**Table 1.**
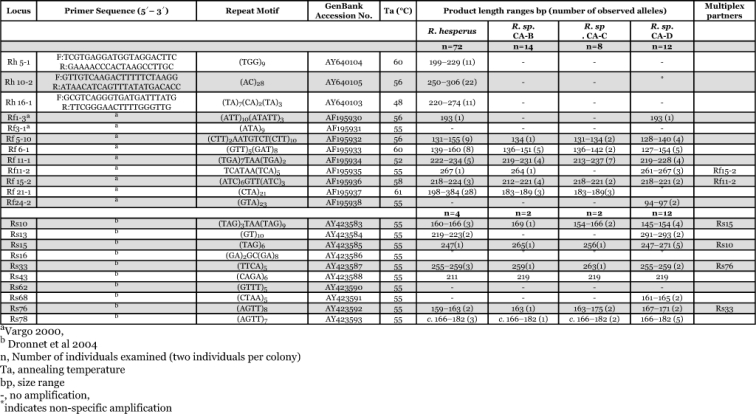
Characteristics of 22 microsatellite loci and cross-amplification results for four western North American *Reticulitermes* species.

In the state of California, *R. hesperus* is the most common subterranean termite. It is found sympatrically in northern California with four putative, yet unnamed, sister species: *R. sp.* CA-B, *R. sp.* CA-C, *R. sp.* CA-D. *R. sp.* CA-B and *R. sp.* CA-C are rare compared to *R. hesperus* and *R. sp.* CA-D. In this paper three new microsatellites are described for *R. hesperus*, and the use of primers previously developed for *R. flavipes* and *R. santonensis* with all four putative California species are examined with the goal of finding loci informative for both population and species level studies.

## Materials and Methods

Microsatellites were developed using methods from Toonen (1997). Approximately 2 µg of DNA was extracted using phenol-chloroform methods by pooling DNA from 10 worker termites from the same colony, and digested using Sau3AI. 15ng of 350–850 bp DNA was ligated into pBluescript KS+ vector (Stratagene, www.stratagene.com), and transformed into XL2 Epicurian Coli Ultra competent cells (Stratagene). Approximately 3,000 recombinant clones were lifted onto nylon membranes and were screened by hybridization with two sets of DIG-3′ end-labeled probes [55°C: (AAC)8, (AAG)8, (AAT)8, (ATC)8, (ACT)8, 65°C: (AC)12,(AG)12] (Boehringer- Mannheim, www.roche.com). Plasmids from 22 positive clones were purified using a miniprep kit (Quiagen, www.qiagen.com) and sequenced on an ABI-377XL automated sequencer through the Division of Biological Sciences Core Sequencing Facility at the University of California, Davis. From the 22 positive clone sequences, seven sets of primers were designed using Primer 3 Software (Rozen and Skaletsky 1996), three of which were polymorphic and deemed acceptable for population genetic studies. These three primer pairs (Rh5-1, Rh10-2, Rh16-1) plus nine sets of primers (Rf) from Vargo (2000) and ten (Rs) from Dronnet et al. 2004 were screened for variability.

**Table 2.**
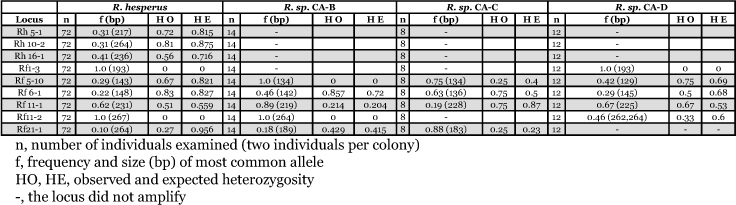
Genetic diversity of selected loci in four western North American *Reticulitermes* species

**Table 3.**
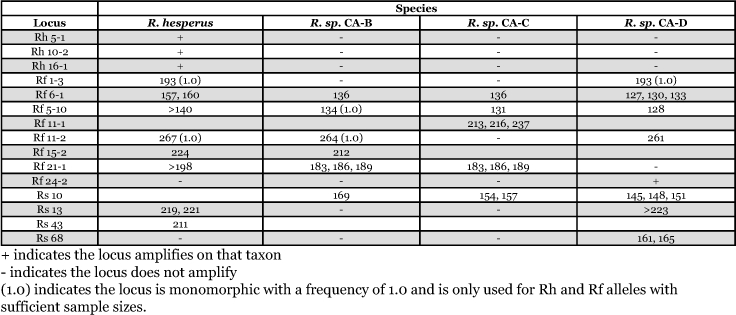
Loci appropriate for taxonomic studies. The table identifies non-overlapping alleles unique to one or two taxa per locus and amplification differences.

Each locus was screened by genotyping two individuals per colony. Screening of colonies was focused on *R. hesperus* and *R. sp.* CA-D as these two species have the largest distribution in California and are the most common taxa found. *R. sp.* CA-B and *R. sp.* CA-C have been reported from a small area and colonies can be difficult to find (Copren et al. 2005). More colonies were screened for Rh and Rf loci than Rs loci because of the likelihood of finding more variation with 2–3bp repeats than the 4bp repeats found in the majority of Rs loci (Dronnet et al. 2004). Thirty-six colonies of *R. hesperus* were examined, as well as four to seven colonies from the other species for the Rh and Rf primers. One to six colonies were screened of each taxon for the Rs primers (Table 1). All available colonies were used for screening, but where few colonies were available, estimates of polymorphism were limited to only those loci with sufficient samples.

Nine individuals from three colonies were gutted and tested on all Rh loci. Amplification of the loci was tested on individuals with the mid- and hindguts removed. The loci amplified successfully indicating that contamination of gut microbes was not a problem. Therefore, whole bodies were then extracted using a standard phenol-chloroform protocol for further analyses. Rh and Rf primers were analyzed using manual sequencing techniques for fragment analysis (Gibco BRL, www.lifetech.com). PCR reactions were set up in 25 µl reaction volumes containing 5 µg of DNA, 1.5mM MgCl2, PCR buffer (50 mM KCL, 10mM tris, 0.1% gelatin, 0.1% Triton-X), 0.2 mM each dNTP, 0.5U Taq polymerase, and 0.5 mM forward and reverse primers. PCR was performed on a PTC-200 thermal cycler (MJ Research, www.mjr.com) using the following program: initial denaturation step 94°C (2 min), 30 cycles at 94°C (30 sec), primer Tm (Table 1, 30 sec), 72°C (30 sec), followed by a final extension step of 7 min at 72°C. PCR products were resolved on a 6% denaturing polyacrylamide gel and visualized using silver. Sizing of alleles at each locus was done manually by comparison to a sequenced pGEM -3Zf(+) plasmid vector (Promega, www.promega.com) and previously scored alleles.

Rs primers were screened using automated sequencing techniques for fragment analysis and primers were end-labeled with a 5′IRD800 fluorescent modification (MWG-Biotech, www.mwgdna.com). PCR amplifications were performed as described in Dronnet et al. (2004). PCR products were separated by electrophoresis on 6% polyacrylamide gels and run on a Li-Cor (www.licor.com) 4000 L DNA sequencer. Alleles were scored using Geneprofiler 4.0.3 software (Scanalytics, www.scanalytics.com).

## Results and Discussion

The loci studied in this paper show variability appropriate for both population and species level studies. The three loci specifically developed for *R. hesperus* did not amplify in any of the other species. Of the 19 remaining loci, 14 gave scoreable products for *R. hesperus*, 12 for *R. sp.* CA-B, 11 *for R. sp.* CA-C, and 15 for *R. sp.* CA-D (Table 1). Of all loci, 13 were polymorphic in *R. hesperus*, 4 in *R. sp.* CA-B, 8 in *R. sp.* CA-C, 13 in *R. sp*., CA-D indicating these loci would have variation appropriate for population level studies (Table 1). Levels of heterozygosity of Rh and Rf loci reported in Table 2 range from a low of 0.21 for locus Rf 11-2 in *R. sp.* CA-B to a high of 0.96 for locus Rf 21-1 in *R. hesperus*, further supporting their utility in population studies.

If all four putative species were synonymous, then patterns of microsatellite amplification would be similar. However, results from 15 loci showed alleles or amplification patterns unique to a minimum of one or two species and should be useful for taxonomic studies among the 4 species (Table 3). Four loci only amplify in one species (Rh 5-1, Rh 10-2, Rh 16-1 in *R. hesperus*; Rf 24-2 and Rs 68 in *R. sp.* CA-D). Loci Rf 5-10 and Rf 11-2 have alleles unique to each of the four species. *R. sp.* CA-B is monomorphic at Rf5-10, and *R. sp.* CA-C has unique alleles at Rf11-1. Specifically, finding fixed genetic differences among cryptic species that result in reciprocal monophyly, particularly among sympatric species, will provide information useful for taxonomic studies.
